# DPM-UNet: A Mamba-Based Network with Dynamic Perception Feature Enhancement for Medical Image Segmentation

**DOI:** 10.3390/s25227053

**Published:** 2025-11-19

**Authors:** Shangyu Xu, Xiaohang Liu, Hongsheng Lei, Bin Hui

**Affiliations:** 1 Key Laboratory of Opto-Electronic Information Processing, Chinese Academy of Sciences, Shenyang 110016, China; 2 Shenyang Institute of Automation, Chinese Academy of Sciences, Shenyang 110016, China; 3 University of Chinese Academy of Sciences, Beijing 100049, China

**Keywords:** medical image segmentation, Mamba, local global feature fusion, multi-scale feature

## Abstract

In medical image segmentation, effective integration of global and local features is crucial. Current methods struggle to simultaneously model long-range dependencies and fine local details. Convolutional Neural Networks (CNNs) excel at extracting local features but are limited by their local receptive fields for capturing long-range dependencies. While global self-attention mechanisms (e.g., in Transformers) can capture long-range spatial relationships, their quadratic computational complexity incurs high costs for high-resolution medical images. To address these limitations, State Space Models (SSMs), which maintain linear complexity while effectively establishing long-range dependencies, have been introduced to visual tasks. Leveraging the advantages of SSMs, this paper proposes DPM-UNet. The network employs a Dual-path Residual Fusion Module (DRFM) at shallow layers to extract local detailed features and a DPMamba Module at deep layers to model global semantic information, achieving effective local global feature fusion. A Multi-scale Aggregation Attention Network (MAAN) is further incorporated to enhance multi-scale representations. The proposed method collaboratively captures local details, long-range dependencies, and multi-scale information in medical images. Experiments on three public datasets demonstrate that DPM-UNet outperforms existing methods across multiple evaluation metrics.

## 1. Introduction

Medical image segmentation plays a pivotal role in modern clinical practice and scientific research, with its value extending across the entire workflow from auxiliary diagnosis and treatment planning to therapeutic outcome evaluation [1,2,3,4]. Traditional segmentation methods heavily rely on manual annotation by physicians, which are time-consuming, labor-intensive, inefficient, and suffer from significant inter-observer variability, directly impacting diagnostic consistency and result reproducibility [5,6]. Against this backdrop, deep learning-based automated segmentation techniques have emerged and have been successfully applied to various imaging segmentation tasks, including magnetic resonance imaging (MRI), nuclei segmentation in microscopic images [7,8], and multi-organ segmentation in computed tomography (CT) [9,10]. These techniques, by virtue of their high efficiency, high accuracy, and robust stability [11,12], lay the groundwork for translating precision medicine from concept to clinical application.

Despite significant progress in deep learning for medical image segmentation, effectively integrating local features with global long-range dependencies to further enhance segmentation accuracy remains a key research challenge. Convolutional neural networks (CNNs) [13,14], exemplified by U-Net [15], SegResNet [16], and nnU-Net [17], excel at extracting local detailed features. However, their inherent local receptive fields limit the model’s capacity to capture information from distant regions within an image, thereby constraining further improvements in segmentation accuracy. In contrast, Vision Transformers (ViTs) [18,19], through their global self-attention mechanism, empower each image patch (token) to attend to all other patches, demonstrating superior performance in modeling long-range feature interactions and capturing global context [20,21]. Nevertheless, the computational complexity of the attention mechanism in ViTs grows quadratically with the number of image patches [22], imposing a substantial computational burden when processing high-resolution medical images.

In recent years, State Space Sequence Models (SSMs) [23,24] have garnered significant attention in the field of computer vision due to their efficiency in processing long sequences. Among them, the Mamba model [22], with its linear computational complexity and advantages in global modeling [25,26], has emerged as an effective solution for capturing long-range dependencies in visual tasks. Compared to Transformer architectures, which exhibit quadratic computational complexity, Mamba-based vision models can process long sequences with near-linear computational overhead, significantly enhancing their scalability and practicality in high-resolution image tasks. Vmamba [25] effectively addressed the mismatch between the 1D sequential processing of traditional SSMs and the 2D spatial structure of images by introducing strategies such as image patching, sequence flattening, and cross-scanning, thereby achieving efficient modeling of the global image context. This progress has spurred the development of a series of Mamba-based medical image segmentation models, including U-Mamba [27], SegMamba [28], and SwinUMamba [29]. However, existing models, while achieving global perception, fail to adequately integrate local detailed features and spatial contextual information, which limits their expressive power in complex medical image segmentation tasks.

To fully integrate local and global features, this paper proposes a Mamba-based DPM-UNet network. The network employs a Dual-path Residual Fusion Module (DRFM) at shallow layers to extract low-level visual features, while a DPMamba Module is introduced at deep layers. The DPMamba Module utilizes Mamba to flatten the image into a sequence, capturing long-range dependencies and global contextual information. To further enhance the model’s ability to extract and fuse image features at a low computational cost, a Dynamic Perception Feature Enhancement Block (DPFE) is applied to the globally aware feature maps generated by Mamba. Additionally, we design a Multi-scale Aggregation Attention Network (MAAN) to extract multi-scale information from the outputs of the DRFM and optimize feature transmission along the encoder-to-decoder path via skip connections. The main contributions of this paper are summarized as follows:

(1) We propose a novel segmentation network named DPM-UNet, which integrates the local feature extraction capability of CNNs with the global information aggregation ability of Mamba, aiming to achieve precise medical image segmentation.
(2) We design three key components: the Dual-path Residual Fusion Module (DRFM), the DPMamba Module, and the Multi-scale Aggregation Attention Network (MAAN). The DRFM enhances local feature extraction by fusing features from standard and dilated convolutions. The DPMamba Module leverages Mamba to generate global features and further enhances feature representation in critical channels through a Dynamic Perception Feature Enhancement Block (DPFE). Additionally, the MAAN is embedded in skip connection paths to optimize the transmission and fusion of multi-scale information.
(3) Experimental results on three public medical image segmentation datasets demonstrate that DPM-UNet achieves state-of-the-art segmentation performance compared to existing methods, fully validating the effectiveness and strong generalization capability of our approach for medical image segmentation tasks.

## 2. Methods

**Figure 1:**
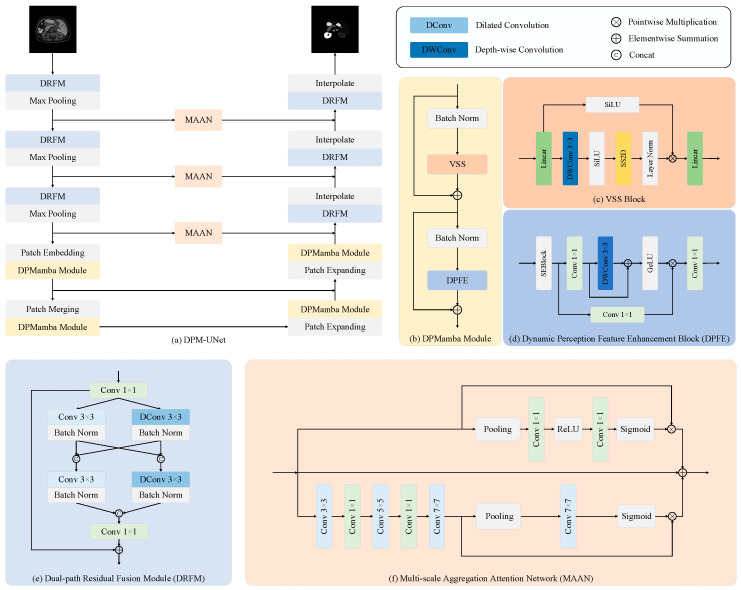
The overall architecture of the proposed Mamba-based U-Net with Dynamic Perception Feature Enhancement (DPM-UNet) for medical image segmentation (DPM-Unet). (**a**) The main framework of DPM-Unet. (**b**) The detailed design of the DPMamba Module, which internally contains a (**c**) VSS Block and a (**d**) Dynamic Perception Feature Enhancement (DPFE) Block. (**e**) The Dual-path Residual Fusion Module (DRFM). (**f**) The Multi-scale Aggregation Attention Network (MAAN).

The overall architecture of DPM-UNet is illustrated in Figure 1a. In contrast to the nearly pure VSS block design of Swin-UMamba, DPM-UNet adopts a hybrid structure: its shallow stages (1–3) use convolution-based DRFMs to focus on extracting local detailed features, while the deeper stages (4–5) incorporate DPMamba modules (VSS-based) to capture global semantic dependencies. The network follows a U-shaped symmetric layout, with max pooling for downsampling and bilinear interpolation for upsampling. Skip connections are incorporated between each corresponding encoder and decoder stage to fuse features across different hierarchies. Additionally, a Multi-scale Aggregation Attention Network (MAAN) is embedded in the skip connections from Stages 1 to 3 to extract multi-scale features and optimize feature transmission during the skip-connection process. This hybrid design balances local and global feature learning, improving performance while reducing complexity compared to pure VSS-based approaches.

### 2.1. DPMamba Module

As shown in Figure 1b, the DPMamba Module first utilizes a VSS Block at the input stage to capture global feature information, and then employs a DPFE Block to further enhance its feature representation capability. Assuming the input feature $X^{l}$ has a shape of $R^{C\times H\times W}$, we have:

(1) $$\;X^{l+1}=VSS\left(BatchNorm\left(X^{l}\right)\right)+X^{l}$$

(2) $$X_{out}=DPFE\left(BatchNorm\left(X^{l+1}\right)\right)+X^{l+1}$$

DPMamba can be decomposed into two independent functional components, VSS(·) and DPFE (·), dedicated to global spatial information extraction and feature refinement, respectively.

#### 2.1.1. VSS Block

Traditional attention mechanisms struggle with efficient long-sequence modeling due to their quadratic computational complexity, posing significant challenges for processing large-scale medical images. The Mamba architecture, based on State Space Sequence Models (SSMs), reduces computational complexity to linear while demonstrating remarkable performance in natural language processing. Leveraging this advantage of Mamba, we introduce its vision variant—the Visual State Space (VSS) model—into the field of medical image segmentation. As illustrated in Figure 1c, the VSS Block employed in this work is designed following the methodology presented in reference [25]. The processing pipeline is as follows: the input features first pass through a linear layer, whose output is split evenly along the channel dimension into two tensors. One branch undergoes processing through depthwise separable convolution, a SiLU activation function, 2D Selective Scanning (SS2D), and layer normalization. The other branch is processed only by a SiLU activation function. Finally, the outputs of the two branches are multiplied element-wise, and the result is fed into another linear layer to produce the final output.

#### 2.1.2. Dynamic Perception Feature Enhancement Block (DPFE)

Recent studies have demonstrated that the gated multilayer perceptron (gated MLP) architecture delivers remarkable performance in natural language processing tasks [30]. We posit that the gating mechanism introduced in this architecture holds significant potential for application in visual tasks as well. Building upon this rationale, we propose the Dynamic Perception Feature Enhancement Block (DPFE), designed to further enhance the model’s capability for image feature extraction and fusion at a low computational cost. The structure of the DPFE module is illustrated in Figure 1d. The output features $X^{l}$ from the VSS Block are first processed by an SE Block [31] for channel-wise attention calibration, yielding features $X^{l+1}$ to emphasize important feature channels while suppressing less significant ones. Subsequently, the features $X^{l+1}$ are split into $X_{1}^{l+1}$ and $X_{2}^{l+1}$, which are fed into two parallel branches. $X_{1}^{l+1}$ passes through a 1 × 1 convolutional layer, followed by a 3 × 3 depthwise separable convolution with residual connections to extract local spatial features, and undergoes non-linear transformation via a GELU activation function, resulting in features $X_{1}^{l+2}$. Meanwhile, $X_{2}^{l+1}$ is processed by a 1 × 1 convolutional layer to produce features $X_{2}^{l+2}$. The features $X_{1}^{l+2}$ and $X_{2}^{l+2}$ are then multiplied element-wise to achieve dynamic weighting based on feature importance. Finally, the weighted result is passed through a 1 × 1 convolutional layer to produce the output $X_{out}$, thereby completing an adaptive feature enhancement process that progresses from channel attention calibration to gated dynamic modulation. The mathematical formulation of the DPFE is as follows:

(3) $$\;X^{l+1}=SE\left(X^{l}\right)$$

(4) $${\;X}_{1}^{l+2}=\sigma \left(f_{3\times 3}^{dec}\left(f_{1\times 1}\left(X_{1}^{l+1}\right)\right)+f_{1\times 1}\left(X_{1}^{l+1}\right)\right)$$

(5) $$\;X_{2}^{l+2}=f_{1\times 1}$$

(6) $$\;X_{out}=f_{1\times 1}\left(X_{1}^{l+2}⊗X_{2}^{l+2}\right)$$

Among these, SE denotes the Squeeze-and-Excitation module, $f_{1\times 1}$ represents a convolutional layer with a kernel size of 1 × 1, $f_{3\times 3}^{dec}$ denotes a depthwise separable convolutional layer with a kernel size of 3 × 3, and *σ*(·) refers to the GELU activation function.

### 2.2. Dual-Path Residual Fusion Module (DRFM)

In medical image segmentation tasks, effectively capturing local features while expanding the receptive field to improve segmentation accuracy remains a core challenge. Existing methods often employ multi-scale convolutional kernels to address this issue. While capable of capturing broader contextual information, these approaches typically incur high computational costs and fail to adequately model the correlations between features under different receptive fields. To overcome these limitations, we propose the Dual-path Residual Fusion Module (DRFM), whose structure is illustrated in Figure 1e. This module adopts a dual-path parallel residual design, synergistically integrating features extracted by standard convolutions and dilated convolutions to generate more informative and robust feature representations. Specifically, the input feature $X^{l}$ first undergoes a 1 × 1 convolution for channel dimension adjustment, yielding feature $X^{l+1}$. It is then fed into two parallel paths: one path uses a 3 × 3 standard convolution to capture local detailed textures, while the other employs a 3 × 3 dilated convolution with a dilation rate of 2 to acquire wide-range contextual information at a lower computational cost. The outputs from both paths are fused to obtain $X^{l+2}$. This feature $X^{l+2}$ subsequently undergoes deeper processing via a parallel set of standard and dilated convolutions, producing $X^{l+3}$. Finally, a 1 × 1 convolution integrates all learned features, and the result is added to the residual connection-provided $X^{l+1}$ to produce the final output feature $X_{out}$. The mathematical formulation of the DRFM is as follows:

(7) $$X^{l+1}=f_{1\times 1}\left(X^{l}\right)$$

(8) $${\;X}^{l+2}=Concat\left[BatchNorm\left(f_{3\times 3}\left(X^{l+1}\right)\right),\;BatchNorm\left(f_{3\times 3}^{2}\left(X^{l+1}\right)\right)\right]$$

(9) $$\;X^{l+3}=Concat\left[BatchNorm\left(f_{3\times 3}\left(X^{l+2}\right)\right),\;BatchNorm\left(f_{3\times 3}^{2}\left(X^{l+2}\right)\right)\right]$$

(10) $${\;X}_{out}=X^{l+1}+f_{1\times 1}\left(X^{l+3}\right)$$

Among these, $f_{1\times 1}$ and $f_{3\times 3}$ denote convolutional layers with kernel sizes of 1 × 1 and 3 × 3, respectively, $f_{3\times 3}^{2}$ represents a dilated convolutional layer with a dilation rate of 2 and a kernel size of 3 × 3, and Concat refers to concatenation along the channel dimension.

### 2.3. Multi-Scale Aggregation Attention Network (MAAN)

In medical image segmentation tasks, significant size variations among target structures impose high demands on multi-scale feature extraction capabilities. To integrate multi-scale semantic information and enhance feature representation capacity for targets of different sizes, we design the Multi-scale Aggregation Attention Network (MAAN), whose structure is illustrated in Figure 1f. This module adopts a parallel dual-branch architecture to enhance input features from both channel and spatial dimensions, respectively. The feature map X output by the DRFM encoder is fed into the MAAN module for refinement. In the channel branch, the spatial dimensions are compressed into channel descriptors of size C × 1 × 1 through average pooling and max pooling. These descriptors then sequentially pass through a 1 × 1 convolution, a ReLU activation function, another 1 × 1 convolution, and a Sigmoid activation function to generate channel attention weights. These weights are multiplied element-wise with the input feature X, yielding the channel-enhanced feature $X_{channel}$. In the spatial branch, the feature X undergoes a serial cascaded structure composed of 3 × 3, 5 × 5, and 7 × 7 convolutions, with 1 × 1 convolutions used for feature aggregation between layers, producing the multi-scale fused feature $X_{MS}$. Subsequently, both average pooling and max pooling are applied, and the resulting features are concatenated along the channel dimension. This is followed by a 7 × 7 convolution and a Sigmoid activation function to generate a spatial attention map. Finally, this map is weighted and fused with $X_{MS}$ to produce the spatially enhanced feature $X_{spatial}$. The channel feature $X_{channel}$ and the spatial feature $X_{spatial}$ are combined with the original input feature X via residual connection to produce the output feature $X_{out}$, thereby enhancing the feature representation capacity in both spatial and channel dimensions. This cross-scale information interaction mechanism effectively improves the model’s ability to represent multi-target structures with significant scale variations. The mathematical formulation of the MAAN is as follows:

(11) $$X_{channel}=\sigma \left(f_{1\times 1}\left(ReLU\left(f_{1\times 1}\left({MP}_{s}\left(X\right)+{AP}_{s}\left(X\right)\right)\right)\right)\right)⊗X$$

(12) $$\;X_{MS}=f_{7\times 7}\left(f_{1\times 1}\left(f_{5\times 5}\left(f_{1\times 1}\left(f_{3\times 3}\left(X\right)\right)\right)\right)\right)$$

(13) $${\;X}_{spatial}=\sigma \left(f_{7\times 7}\left(Concat\left[MP_{c}\left(X_{MS}\right),AP_{c}\left(X_{MS}\right)\right]\right)\right)⊗X_{MS}$$

(14) $${\;X}_{out}=X+X_{channel}+X_{spatial}$$

Among these, $f_{1\times 1}$, $f_{3\times 3}$, $f_{5\times 5}$ and $f_{7\times 7}$ denote convolutional layers with kernel sizes of 1 × 1, 3 × 3, 5 × 5 and 7 × 7; ${MP}_{c}$ and ${AP}_{c}$ indicate max pooling and average pooling operations along the channel dimension; ${MP}_{s}$ and ${AP}_{s}$ represent max pooling and average pooling operations along the spatial dimensions; *σ*(·) denotes the Sigmoid activation function; and Concat refers to concatenation along the channel dimension.

## 3. Experiments

### 3.1. Datasets

(1) The Abdomen MRI dataset: This dataset used in this study is sourced from the publicly available MICCAI 2022 AMOS Challenge resources [29]. It is designed for segmenting 13 abdominal organs, such as the liver, spleen, pancreas, kidneys, stomach, gallbladder, esophagus, aorta, inferior vena cava, adrenal glands, and duodenum. To ensure statistical reliability, the data partition (60 scans for training, 50 for testing) follows the established benchmark protocol in reference [27], which introduced additional scans to overcome limitations of a smaller validation set. These test scans are fully independent, with no patient overlap against the training set, and were annotated by professional radiologists to ensure quality. Throughout the experiments, all images were preprocessed to a resolution of 320 × 320 pixels.

(2) The Microscopy dataset: This dataset focuses on cell instance segmentation tasks, with image data originating from the publicly available NeurIPS 2022 Cell Segmentation Challenge dataset [32]. Its specific composition includes 1000 images for training and 101 images for testing. To standardize the input size, all images were cropped to 512 × 512 pixels before training and testing. The data processing methodology follows the scheme proposed in reference [27].

(3) The ACDC dataset: This dataset comprises cardiac MRI scans from 150 patients, with each patient containing scans from different physiological phases, such as systole and diastole. Sourced from the Automatic Cardiac Diagnosis Challenge [33], its core task is to segment the left ventricle, right ventricle, and myocardium. Our experiment utilizes data from 100 patients in this dataset. We divided these 100 cases into training and test sets in an 8:2 ratio. The training set contains 160 scans from 80 patients, and the test set contains 40 scans from 20 patients. All images used for training and testing were preprocessed and uniformly resized to 256 × 256 pixels.

### 3.2. Evaluation Metrics and Baselines

For MRI-based datasets, Abdomen MRI and ACDC, the Dice Similarity Coefficient (DSC) was used to measure volumetric overlap between segmentations and ground truth, while the Normalized Surface Distance (NSD) evaluated boundary accuracy. For the Microscopy dataset, which involves instance-level cell identification, the F1-Score served as the primary metric to assess detection performance at the object level. A prediction was counted as a True Positive only if the IoU with the ground truth exceeded 0.5. Additionally, the DSC metric was applied to complement this by evaluating pixel-level segmentation accuracy within each correctly detected instance. The calculation formulas for all metrics are as follows:

(15) $$DSC=\frac{2TP}{\left(TP+FN\right)+\left(TP+FP\right)}$$

(16) $$\;NSD=\frac{\left|S_{pred}\cap S_{gt,\tau }\right|+\left|S_{gt}\cap S_{pred,\tau }\right|}{\left|S_{pred}\right|+\left|S_{gt}\right|}$$

(17) $$IoU=\frac{TP}{TP+FP+FN}$$

(18) $$F1=2\times \frac{Precision\times Recall}{Precision+Recall},\;if\;IoU>0.5$$

(19) $$Precision=\frac{TP}{TP+FP}$$

(20) $$Recall=\frac{TP}{TP+FN}$$

Here, TP (True Positive) denotes the number of samples correctly predicted as positive; FP (False Positive) denotes the number of samples incorrectly predicted as positive; and FN (False Negative) denotes the number of samples incorrectly predicted as negative. *S*_*pred*_ and *S*_*gt*_ represent the sets of surface points corresponding to the predicted segmentation and the ground truth segmentation, respectively. *S*_*pred*,*τ*_ is defined as the set of all points in the predicted surface whose distance to the ground truth surface is less than the threshold *τ*; similarly, *S*_*gt*,*τ*_ refers to the set of all points in the ground truth surface whose distance to the predicted surface is within *τ*.

We compared five medical image segmentation models under uniform experimental conditions, covering three major architecture types: The traditional CNN architectures are represented by nnU-Net [17] and SegResNet [16]. The Transformer-based architectures are represented by UNETR [34] and SwinUNETR [35]. Swin-UMamba [29] was selected as the representative Mamba-based architecture. All models were rigorously reproduced within the nnU-Net framework, strictly adhering to the hyperparameter configurations reported in their respective original publications.

### 3.3. Implementation Details

**Figure 2:**
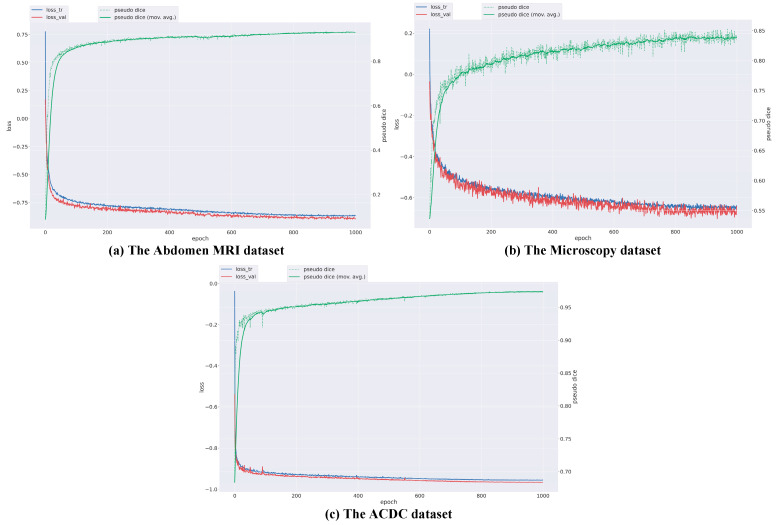
Training loss curves for the three datasets.

In the architecture of the DPM-UNet network, each of stages 4 to 5 is configured with four consecutive DPMamba modules, forming a [4, 4] structure. All models are implemented within the nnU-Net framework without using any pre-trained weights, and all parameters are optimized through training from scratch. This design enables a focused investigation into network architecture innovation while maintaining consistent experimental conditions—such as image preprocessing and data augmentation—across all compared methods. Consequently, DPM-UNet is evaluated under uniform settings, ensuring that the network architecture remains the sole differentiating factor. During training, patch size, batch size, and network configuration adhere to the standard nnU-Net settings. The training process employs the AdamW optimizer with a weight decay coefficient of 0.05. The initial learning rate is set to 2 × 10^−4^ and decays to a minimum of 1 × 10^−6^ using a cosine annealing scheduling strategy. The loss function is defined as the unweighted sum of Dice loss and cross-entropy loss. A five-fold cross-validation strategy is applied on the training set for all three datasets. Since the Dice Similarity Coefficient (DSC) is the most critical evaluation metric in our task, the model checkpoint achieving the highest DSC on the validation set is selected as the optimal model for each training run. The models are trained for 1000 epochs in total without adopting an early stopping strategy, leveraging the cosine annealing learning rate scheduler for full-cycle optimization. The batch size is set to 4 for the microscopy dataset and 8 for the other two datasets. The training curves of three datasets are shown in Figure 2. All experiments are implemented using the PyTorch (torch = 2.1.1) framework on hardware equipped with an NVIDIA RTX 4070 GPU.

### 3.4. Experimental Results

**Figure 3:**
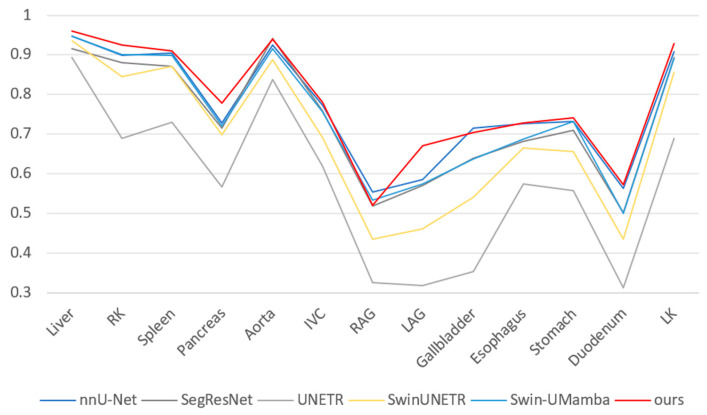
Segmentation Results (DSC) for Various Organs on the Abdomen MRI dataset.

**Figure 4:**
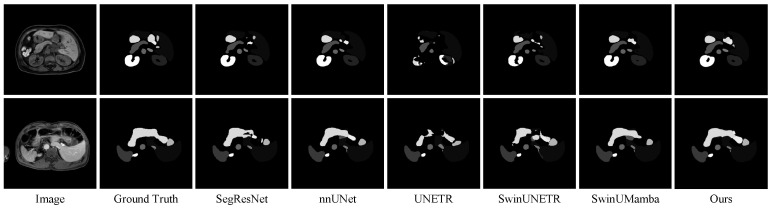
Visualizations on the Abdomen MRI dataset.

**Figure 5:**
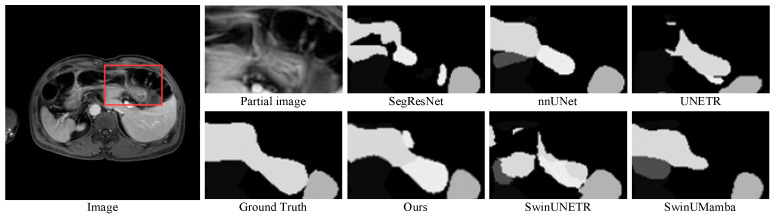
Enlarged view of the region indicated by the red box in the Abdomen MRI visualization.

Table 1 presents the quantitative results of multi-organ segmentation on the Abdomen MRI dataset. The DPM-UNet model achieved a DSC of 78.15%, representing a 1.52 percentage point improvement over the second-best performing nnU-Net, while its NSD reached 84.67%, with an improvement of 1.16 percentage points. As shown in Figure 3, regarding organ-level segmentation accuracy, DPM-UNet ranked first in 10 organs including the liver, left and right kidneys, spleen, pancreas, inferior vena cava, left adrenal gland, esophagus, stomach, and duodenum. The overall segmentation visualization in Figure 4 and the enlarged local details in Figure 5 consistently demonstrate that DPM-UNet not only accurately captures the main structures of organs but also produces more continuous and smoother segmentation contours along complex anatomical boundaries. These results fully validate the effectiveness of the proposed global–local feature collaboration mechanism in representing multi-scale anatomical structures.

**Figure 6:**
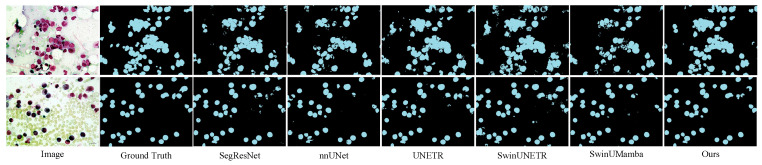
Visualizations on the Microscopy dataset.

**Figure 7:**
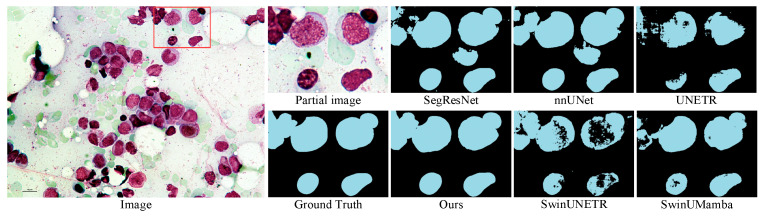
Enlarged view of the region indicated by the red box in the Microscopy visualization.

The experimental results on the Microscopy dataset are presented in Table 2. The DPM-UNet model achieved a DSC of 73.25%, representing a 1.56 percentage point improvement over the second-best performing UNETR, while its F1-score reached 60.23%, with an improvement of 5.58 percentage points. As shown in the visual comparison in Figure 6, DPM-UNet accurately captures the main structures of cells. The enlarged local details in Figure 7 further demonstrate that DPM-UNet effectively identifies and separates small-sized or adherent nuclei with more precise edge delineation. These results underscore the advantages of the model’s local feature extraction mechanism, which enables it to capture subtle differential features and fine boundary information between nuclei, thereby significantly improving segmentation accuracy.

**Figure 8:**
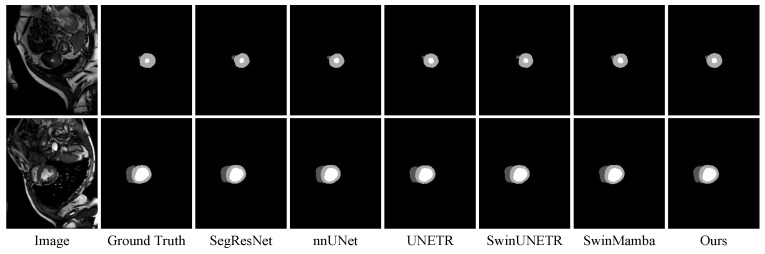
Visualizations on the ACDC dataset.

**Figure 9:**
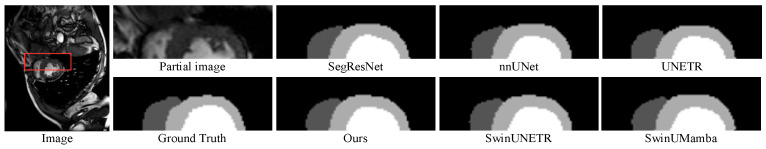
Enlarged view of the region indicated by the red box in the ACDC visualization.

The experimental results on the ACDC dataset are presented in Table 3. While the DPM-UNet model achieved optimal performance, the overall performance gap among different models was relatively small. Specifically, DPM-UNet improved the DSC metric by 0.14% compared to nnU-Net and enhanced the NSD metric by 0.05% compared to Swin-UMamba. In the substructure segmentation tasks, DPM-UNet delivered optimal performance for myocardial (Myo) and left ventricular (LV) segmentation, while ranking third in right ventricular (RV) segmentation. The overall comparison in Figure 8 and the enlarged local details in Figure 9 demonstrate that DPM-UNet generates more accurate and sharper segmentation contours, further validating its local–global feature interaction capability in effectively capturing critical subtle structural features along the edges.

### 3.5. Further Analysis

#### 3.5.1. Ablation Study

The ablation experiments on the abdominal MRI dataset evaluated the effectiveness of the proposed modules, with results summarized in Table 4. The introduction of the DRFM alone improved performance, increasing the Dice Similarity Coefficient (DSC) by 0.45% and the Normalized Surface Distance (NSD) by 0.88%. After integrating both the DRFM and DPMamba modules, the model performance was further enhanced, achieving additional gains of 0.40% in DSC and 0.41% in NSD. This demonstrates the capability of the DPMamba module to effectively improve segmentation accuracy. Subsequently, incorporating the CBAM module led to further improvements, with DSC and NSD increasing by an additional 0.82% and 0.73%, respectively. When the CBAM module was replaced with the MAAN module, the model achieved its optimal performance, reaching a DSC of 78.15% and an NSD of 84.67%. MAAN outperforms CBAM due to its structural advantage; a dual-path design that concurrently models’ channel and spatial relationships, combined with multi-scale feature aggregation, which enables more effective context capture than CBAM’s sequential design. These results fully validate the effectiveness of the designed modules and their synergistic contribution to enhancing the model’s overall segmentation capability. (**Note**: In the following table, a check mark (✔) or cross mark (✘) indicates the presence or absence of the corresponding module. An upward (↓) or downward (↑) arrow indicates that a higher or lower value denotes better performance, respectively.)

**Figure 10:**
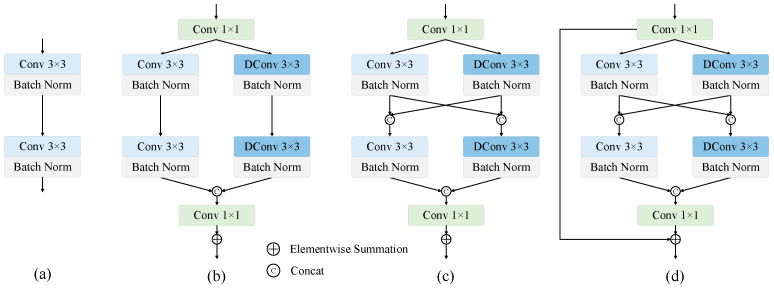
Comparison of convolutional module structures. (**a**) Standard convolution block, (**b**) Dual-path convolution block, (**c**) Dual-path convolution block with feature mixing, (**d**) Our proposed DRFM block.

To further investigate the impact of the DRFM structure on the model’s feature extraction capability, we conducted additional ablation experiments, with the results summarized in Table 5. The corresponding schematic diagrams of each experimental setup can be found in Figure 10a–d. The experimental results demonstrate that introducing dilated convolutions in the second path effectively enhances model performance. Furthermore, fusing features extracted by standard convolutions and dilated convolutions yielded more substantial performance improvements. Additionally, the incorporation of residual connections also contributed significantly to the overall performance enhancement. These results indicate that the DRFM can effectively strengthen the model’s feature representation capability.

#### 3.5.2. Model Complexity

As shown in Table 6, this study employs floating-point operations (FLOPs), number of parameters (Params), and the total training time over 1000 epochs as key metrics to evaluate model complexity and practical training efficiency. In terms of computational and storage complexity, DPM-UNet demonstrates a moderate level among the compared models. However, its training time is the second longest, indicating a higher computational cost during the training phase. Critically, the superior performance of DPM-UNet justifies this increased training cost. It achieves the highest Dice Similarity Coefficient (DSC) and Normalized Surface Dice (NSD), demonstrating that the method achieves a favorable balance between computational efficiency and segmentation accuracy.

## 4. Conclusions and Future Work

This study proposes DPM-UNet, a medical image segmentation network based on the Mamba architecture. The method adopts a U-shaped structure, where a Dual-path Residual Fusion Module (DRFM) is introduced in the shallow layers to enhance local detail feature extraction, while a DPMamba module capable of modeling long-range dependencies is employed in the deep layers to capture global contextual information. Furthermore, the Multi-scale Aggregation Attention Network (MAAN) is incorporated to strengthen the model’s ability to perceive and fuse multi-scale features. Experimental results on three public medical image segmentation datasets demonstrate the superior segmentation performance of the proposed method. While the current study has established a solid foundation, certain aspects, such as multi-run performance validation, statistical significance testing, and detailed efficiency profiling, were not fully explored within the scope of this work. These areas present meaningful opportunities for further investigation. Future efforts will focus on developing more lightweight architectures and efficient training strategies to reduce computational costs, alongside incorporating rigorous benchmarking and efficiency analysis to enhance the robustness and practicality of the model under resource constraints.

## Figures and Tables

**Table 1:** Segmentation accuracy of different models on the Abdomen MRI dataset. The best results are displayed in bold, and the second-best results are indicated with an underscore.

Methods	DSC	NSD
nnU-Net	76.63	83.51
SegResNet	73.84	80.13
UNETR	57.46	62.82
SwinUNETR	69.08	74.73
Swin-UMamba	74.56	81.15
DPM-UNet	**78.15**	**84.67**

**Table 2:** Segmentation accuracy of different models on the Microscopy dataset. The best results are displayed in bold, and the second-best results are indicated with an underscore.

Methods	DSC	F1
nnU-Net	69.55	54.65
SegResNet	68.51	54.02
UNETR	71.69	40.57
SwinUNETR	66.69	37.08
Swin-UMamba	67.60	49.00
DPM-UNet	**73.25**	**60.23**

**Table 3:** Segmentation accuracy of different models on the ACDC dataset. The best results are displayed in bold, and the second-best results are indicated with an underscore.

Methods	DSC	NSD	RV	Myo	LV
nnU-Net	91.81	97.88	89.44	90.60	95.38
SegResNet	91.71	97.99	89.64	90.27	95.23
UNETR	89.34	95.49	86.76	87.46	93.80
SwinUNETR	91.50	97.61	89.41	90.03	95.06
Swin-UMamba	91.69	98.04	**90.00**	89.78	95.30
DPM-UNet	**91.95**	**98.09**	89.46	**90.76**	**95.64**

**Table 4:** Ablation study of the designed blocks on the Abdomen MRI dataset.

DRFM	DPMamba	CBAM	MAAN	DSC ↑	NSD ↑
✘	✘	✘	✘	75.17	81.52
✔	✘	✘	✘	75.62	82.40
✔	✔	✘	✘	76.02	82.81
✔	✔	✔	✘	76.84	83.54
✔	✔	✘	✔	78.15	84.67

**Table 5:** Ablation study of DRFM structure on the Abdomen MRI dataset.

Dilated Convolution	Fusion	Residual	DSC ↑	NSD ↑
✘	✘	✘	76.04	82.80
✔	✘	✘	76.35	83.06
✔	✔	✘	77.53	84.32
✔	✔	✔	78.15	84.67

**Table 6:** Comparison of FLOPs and Params on the Abdomen MRI dataset using our method with other models.

Methods	FLOPs (G) ↓	Param. (M) ↓	Training Time (H)	DSC ↑	NSD ↑
nnU-Net	23	33	6	76.63	83.51
SegResNet	24	6	8	73.84	80.13
UNETR	41	87	17	57.46	62.82
SwinUNETR	29	25	20	69.08	74.73
Swin-UMamba	63	59	30	74.56	81.15
DPM-UNet	31	38	24	78.15	84.67

## Data Availability

You can download the AbdomenMRI/Microscopy/ACDC dataset at https://drive.google.com/drive/folders/1CH2OWQpd4Sa-BES6oFLRC469gTxf6QUO (accessed on 11 November 2025).
